# Lysosomal Two-pore Channel Subtype 2 (TPC2) Regulates Skeletal Muscle Autophagic Signaling*

**DOI:** 10.1074/jbc.M114.608471

**Published:** 2014-12-05

**Authors:** Pei-Hui Lin, Pu Duann, Shinji Komazaki, Ki Ho Park, Haichang Li, Mingzhai Sun, Mathew Sermersheim, Kristyn Gumpper, John Parrington, Antony Galione, A. Mark Evans, Michael X. Zhu, Jianjie Ma

**Affiliations:** From the ‡ Department of Surgery, Davis Heart and Lung Research Institute, The Ohio State University Wexner Medical Center, Columbus, Ohio 43210,; § Department of Anatomy, Saitama Medical University, Saitama 350-0495, Japan,; ¶ Department of Pharmacology, University of Oxford, Oxford OX1 3QT, United Kingdom,; ‖ Centre for Integrative Physiology, College of Medicine and Veterinary Medicine, University of Edinburgh, Edinburgh EH8 9XD, Scotland, United Kingdom, and; ** Department of Integrative Biology and Pharmacology, The University of Texas Health Science Center, Houston, Texas 77030

**Keywords:** Autophagy, Calcium Channel, Lysosome, Muscle Atrophy, Protein Turnover

## Abstract

Postnatal skeletal muscle mass is regulated by the balance between anabolic protein synthesis and catabolic protein degradation, and muscle atrophy occurs when protein homeostasis is disrupted. Autophagy has emerged as critical in clearing dysfunctional organelles and thus in regulating protein turnover. Here we show that endolysosomal two-pore channel subtype 2 (TPC2) contributes to autophagy signaling and protein homeostasis in skeletal muscle. Muscles derived from *Tpcn2*^−/−^ mice exhibit an atrophic phenotype with exacerbated autophagy under starvation. Compared with wild types, animals lacking TPC2 demonstrated an enhanced autophagy flux characterized by increased accumulation of autophagosomes upon combined stress induction by starvation and colchicine treatment. In addition, deletion of TPC2 in muscle caused aberrant lysosomal pH homeostasis and reduced lysosomal protease activity. Association between mammalian target of rapamycin and TPC2 was detected in skeletal muscle, allowing for appropriate adjustments to cellular metabolic states and subsequent execution of autophagy. TPC2 therefore impacts mammalian target of rapamycin reactivation during the process of autophagy and contributes to maintenance of muscle homeostasis.

## Teaser

**Background:** The endolysosomal TPC2 ion channel interacts with mTOR to regulate cellular energy utilization.

**Results:** Mice lacking TPC2 display muscle atrophy phenotype with reduced muscle endurance, altered autophagy, and lysosomal enzymatic activities.

**Conclusion:** TPC2 regulates autophagic signaling in skeletal muscle.

**Significance:** TPC2 impacts protein turnover via regulating autophagy signaling in the process of tissue homeostasis and aging.

## Introduction

Skeletal muscle constitutes the largest organ of the mammalian body. In addition to motor control, muscle provides a large reserve of proteins and plays a vital role in regulating electrolyte balance (K^+^ and Ca^2+^), nutrient availability (*e.g.* glucose and amino acids), and pH homeostasis (1). Understanding the mechanisms and factors that regulate protein turnover in skeletal muscle is important for physiology and medicine. Skeletal muscle protein turnover can be mediated via several mechanisms, including ubiquitin/proteasome-dependent and autophagy/lysosome-dependent proteolytic pathways (2). An imbalance between these pathways can lead to muscle atrophy and sarcopenia during aging.

Autophagy is a cytoprotective “self-eating” process utilized by eukaryotic cells and tissues as a defense mechanism against environmental stress, such as disease, starvation, and infection (3, 4). Basal autophagy provides for cellular “quality control” and determines, in part, the turnover of long-lived proteins and the selective degradation of damaged cellular components to maintain tissue homeostasis. Autophagy also plays important roles during development and differentiation (5–7). During this catabolic process, cytoplasmic constituents are sequestered into double membrane-bound vesicles, named autophagosomes, and delivered to lysosomes for degradation and subsequent nutrient regeneration (8). It has now been recognized that autophagy is a multistep process regulated by a complex set of molecular mechanisms that control different steps, such as the formation of the phagophore and then autophagosome, the fusion of autophagosomes with lysosomes to form autolysosomes, and autophagic lysosome reformation (9, 10). The importance of these processes to the regulation of skeletal muscle physiology is now clear (11, 12) as altered lysosomal function has recently been reported in several classes of myopathy (13).

The AKT^3^/mammalian target of rapamycin (mTOR) pathway is pivotal to cell growth, survival, and metabolic regulation through their kinase regulation and the subsequent modulation of numerous downstream substrate activities (14). Cumulative studies have confirmed the complex interplay between the AKT/mTOR pathway, autophagy, and muscle wasting (15, 16). The AKT isoforms undergo phosphorylation-dependent activation and plasma membrane recruitment through their association with phosphatidylinositol 3,4,5-trisphosphate. In addition, the active phosphorylated AKT (p-AKT) has been shown to modulate the activity of FoxO3, a transcription factor that in turn regulates the expression of autophagy and proteasome-related genes in muscle (11, 17). AKT mediates some of its cellular signaling via regulation of mTOR activity, which couples cellular nutrient status to the regulation of systematic growth and metabolism (18, 19). Two distinct complexes, namely mTOR complex 1 and mTOR complex 2, which differ by way of their association with other protein partners and their sensitivity to the allosteric mTOR inhibitor, rapamycin, have been identified (20). Of these, mTOR complex 1 functions as a cellular nutrient sensor that interacts with lysosomes to integrate environmental signals and cellular growth responses by promoting anabolic and suppressing catabolic functions (21).

Recently, it was shown that mTOR interacts with two-pore channel subtypes 1 and 2 (TPC1 and TPC2) and regulates their functions in response to changes in cytosolic ATP levels; the *Tpcn1*/*Tpcn2* double knock-out mice exhibited poor endurance in the treadmill test during starvation (22). These results suggested a role for TPCs in the starvation response and/or autophagy. However, initial analysis in liver, heart, and macrophages of the double knock-out animals did not reveal any detectable defect in autophagy (22). TPCs are endolysosome cation channels that are required for Ca^2+^ release from acidic organelles in response to nicotinic acid adenine dinucleotide phosphate (NAADP) (23, 24) and Na^+^ efflux activated by phosphatidylinositol 3,5-bisphosphate (25) and may also be important for maintaining the proton gradient and membrane potential of endolysosomes (22). This is of particular interest because the NAADP/TPC pathway appears to be tightly associated with autophagy in cell culture models. Overexpression of TPC2 or NAADP agonists has been shown to enhance autophagosome accumulation as indicated by the presence of the lipidated microtubule-associated protein 1A/1B light chain 3A (LC3-II) (26, 27). Moreover, down-regulation of TPC1/TPC2 expression or application of NAADP antagonists reduced LC3-II levels (26–28). That aside, however, we know little of the precise mechanism by which TPC proteins contribute to autophagy *in vivo*.

Here, using a different line of *Tpcn*2 knock-out mice than those used by others (22), we examined the role of *Tpcn*2 in autophagy of skeletal muscles, autophagic response to starvation, and age-dependence of this process. Our results suggest a role of TPC2 in maintaining muscle mass and endurance via regulation of mTOR reactivation and autophagy termination.

## EXPERIMENTAL PROCEDURES

### 

#### 

##### Animals and Treatments

All animal care and usage followed National Institutes of Health guidelines and Institutional Animal Care and Use Committee approval by The Ohio State University. *Tpcn2*^−/−^ mice were derived from GeneTrap mutagenesis as described previously (23). The mice were backcrossed to C57BL6/J (The Jackson Laboratory) for six generations before being used for experiments. The same generations of wild type and *Tpcn2*^−/−^ mice were used for experiments.

For animal autophagy flux measurement, mice (male; 4–5 months old) were subjected to regular chow, 2-day fasting alone, colchicine (0.4 mg/kg/day, Sigma) alone, or a combined treatment of fasting and colchicine treatment. Animals had free access to water during the 2-day starvation period. Colchicine was administered via intraperitoneal injection (29).

For animal dietary states, animals (male; 4–5 months old) were subjected to either regular chow or 3-day fasting (autophagy induction) prior to experimentation. Animals had free access to water during the 3-day starvation period.

For animal endurance assays, animals were first subjected to either regular chow or 3-day fasting with free access to water prior to experimentation. Endurance was assessed with treadmill (model Eco3/6, Columbus Instruments) exercise. Animals were acclimated to the treadmill by running for 5 min at 8 m/min each day for 3 days. On the day of experimentation, the treadmill was adjusted to the running speed of 12 m/min, and an electrical stimulus (163 V, 0.45-mA current, 1 Hz) was created through electrical shock grids at the rear end of the treadmill. Animals were allowed to exercise until exhaustion, and the total distance traveled was recorded. Exhaustion is defined as the animal contacting the shock grid for greater than 10 consecutive seconds without attempting to re-engage the treadmill. To identify TPC2-interacting proteins in skeletal muscle, HA-TPC2 plasmid (23) was electroporated (40 μg of cDNA/tibialis anterior (TA) muscle) and expressed in TA muscle according to our previously published method (30).

##### Histology and Anti-dystrophin Immunohistochemistry

The animals were sacrificed by cervical dislocation, and TA, extensor digitorum longus, soleus, and gastrocnemius (GA) muscles were dissected, weighed, and placed into 10% formalin for 2 days. Tissues were then processed for paraffin embedding and either H&E or trichrome stains (The OSU Pathology Core). Cross-sections of TA muscle were stained with anti-dystrophin to reveal myofiber boundaries. Muscle paraffin sections were deparaffinized, rehydrated, antigen-retrieved, incubated with anti-dystrophin (Spring Bioscience), and developed with the Vectastain ABC (rabbit IgG) kit (Vector Laboratories) followed by H&E counterstaining.

Morphometric analyses were performed on sections collected from similar region of each TA muscle. Ten images were captured from different areas covering the entire TA section with an Olympus DP73 camera attached to a Zeiss Axiovert 200 microscope. Images were analyzed with cellSens Digital Imaging software (Olympus Global) to determine the cross-sectional area from 500–1000 myofibers/animal.

##### Transmission Electron Microscopy (EM)

TA muscles were dissected from hind limbs of adult mice and prepared for EM. Briefly, skeletal muscles were fixed in 3% paraformaldehyde, 2.5% glutaraldehyde, and 0.1 m cacodylate buffer (pH 7.4) and later postfixed in 1% OsO_4_ and 0.1 m cacodylate buffer (pH 7.4). Microthin sections were double stained with uranyl acetate and lead citrate. These sections were examined under a transmission electron microscope (JEM-1010, JEOL, Tokyo, Japan), and the numbers of autophagosomes and lysosomes (based on morphological appearance) were counted.

##### Plasmids

Plasmids for *gfp-tpcn2* and *HA-tpcn2*, which express GFP-TPC2 and HA-TPC2, respectively, were described previously (23). pcms-eGFP plasmid was acquired from Clontech.

##### Western Blotting and Co-immunoprecipitation

Tissue samples from TA muscles were homogenized in a lysis buffer containing 10% SDS, 70 mm Tris-HCl, 10 mm EDTA, and 5% β-mercaptoethanol (pH 6.8) supplemented with a mixture of protease inhibitors (Sigma) and phosphatase inhibitors (Thermo Scientific). Myoblast/myotube protein extraction was performed with modified radioimmune precipitation assay buffer plus the mixture of protease and phosphatase inhibitors. The lysates were separated by SDS-PAGE and analyzed by immunoblotting with appropriate first antibodies and the corresponding species specific HRP-labeled light chain secondary antibodies (Jackson ImmunoResearch Laboratories). Antibodies for Western blotting were as follows: α-tubulin (Sigma), LC3 (clone 5F10, Nanotools), lysosome-associated membrane protein-1 (LAMP1) (Santa Cruz Biotechnology), HA (clone12CA5, Roche Applied Science), dystrophin (Spring Bioscience), p62/sequestosome-1 (SQSTM1) (Novus Biological), S6K, phospho-S6K (phospho-Thr-389), S6RP (ribosomal protein), phospho-S6RP (phospho-Ser-235/236), AKT and phospho-AKT, and mTOR and phospho-mTOR (phospho-Ser-2448) (all from Cell Signaling Technology).

Co-immunoprecipitation assays of HA-TPC2-interacting proteins in TA muscle were performed as follows. Two weeks post-muscle electroporation, TA muscles were homogenized in 0.5 ml of modified radioimmune precipitation assay buffer plus protease inhibitor mixture, and proteins were extracted and precleared with a mixture of protein A- and protein G-agarose (KPL, Kirkegaard and Perry Laboratories, Inc.). Precleared cell lysate was incubated overnight with 10 μg of anti-HA mAb (clone12CA5) or normal mouse IgG (as a negative immunoprecipitation control; from Jackson ImmunoResearch Laboratories). The resulting immunocomplexes were collected on protein A/G-agarose beads and separated by SDS-PAGE.

##### Myoblast Isolation, Transfection, and Myoblast Autophagy Flux Measurement

Primary myoblast isolation and culture were performed according to the method developed previously (31). Limbs derived from 3-day-old wild type or *Tpcn2*^−/−^ pups were digested with a mixture of collagenase D (25 mg/ml; Roche Applied Science) and Dispase (50 units/ml; BD Biosciences) in DMEM for 30 min. The enzymes were removed by centrifugation, and the cell pellets were collected and triturated to suspension in DMEM containing 10% fetal bovine serum (FBS). Cells were then filtered through a cell strainer (70-μm mesh size) to remove large undigested tissue debris and settled in Petri dishes for 2 h for fibroblasts to attach. The non-attached myoblast cell suspension was centrifuged and resuspended in Ham's F-10 medium supplemented with 20% FBS, 100 units/ml penicillin, and 100 μg/ml streptomycin. Cells were grown in a humidified environment at 37 °C and 5% CO_2_. Myotube differentiation was induced by replacing myoblast growth medium with a differentiation medium (DMEM with 5% horse serum).

For *in vitro* myotube autophagic flux analysis, 4-day-differentiated myotubes were subjected to autophagy flux assay as described previously (32). Briefly, myotubes were washed twice with Hanks' balanced saline solution (HBSS; Invitrogen) and then subjected to one of the following four treatments: medium, medium with bafilomycin A1 (Baf A1; 200 nm; Tocris Bioscience, Bristol, UK), HBSS, and HBSS plus Baf A1 for 3.5 h. The concentration of Baf A1 was chosen to ensure the complete block of autophagosome-lysosome fusion. At the end of the incubation, cells pellets were collected and lysed, and the lysates were subjected to Western blot assays for quantification of relative LC3 levels with densitometry analysis using NIH ImageJ software.

To confirm the effect of TPC2 on autophagy flux, the *gfp-tpcn2* or pcms-eGFP plasmid was transfected into *Tpcn2*^−/−^ myoblasts with Lipofectamine LTX reagents (Invitrogen) according to the manufacturer's instructions. After 2 days, the transfected cells were subjected to autophagy flux analysis as described above.

##### Myoblast Lysosomal pH Imaging

Ratiometric lysosome pH measurements with Oregon Green 514-labeled dextran were performed as described previously (33). Wild type and *Tpcn2*^−/−^ myoblasts were cultured on glass bottom ΔT dishes and loaded overnight with pH-sensitive dye Oregon Green 514-labeled dextran (molecular weight, 70,000; 500 μg/ml; Invitrogen) in culture medium. Cells were washed twice and chased (to clear endocytosis) for 3 h at 37 °C in culture medium. Fluorescence video images (excitation wavelengths were 440 and 490 nm; the emission wavelength was 514 nm) were acquired using confocal microscope LSM780. During pH imaging, cells were assayed in culture medium (pH 7.4) first; at the end of imaging each dish, *in situ* pH calibration was performed in isotonic MES buffer (5 mm glucose, 25 mm Na-MES, 1 mm CaCl_2_, 1 mm MgCl_2_, 120 mm KCl, and 20 mm NaCl with pH standards ranging from 7.0 to 3.0 at an interval of 0.5) supplemented with ionophore (5 μm nigericin; Invitrogen). Cells were pre-equilibrated in each pH standard for 5 min to allow pH equilibration across the lysosome membrane and then imaged. The mean fluorescence intensity ratio (*F*_490_/*F*_440_) was calculated from 20 lysosomes/cell/condition for a total of three experimentations. The ratiometric pH curve with Oregon Green 514-dextran fluorescence intensity (*F*_490_/*F*_440_ ratio) as a function of pH was fitted to a Boltzmann sigmoid curve. Lysosome pH values were obtained by fitting the intensity ratios from experiments to the pH standard curves.

##### Skeletal Muscle Acid Enzyme Extraction and Cathepsin Activity Assay

The protocol for cathepsin enzyme kinetics was reported previously (34). In detail, GA muscles were dissected from wild type or *Tpcn2*^−/−^ mice and extracted with 3 volumes of ice-cold cytosol lysis buffer (20 mm Tris-HCl, 1 mm EDTA, 1 mm EGTA, 1% glycerol, and freshly prepared dl-dithiothreitol (DTT) to a final concentration of 2 mm (pH 7.8)) with an OMNI tissue homogenizer (OMNI International). The pellets from centrifugation (13,000 × *g*, 30 min, 4 °C) were further extracted with 2 volumes of acid lysis buffer (200 mm sodium acetate, 50 mm NaCl, and 0.1% Triton X-100 (pH 5.0)), sonicated, and centrifuged again. The soluble lysates (15 μg) were then subjected to cathepsin enzyme kinetics assay with 20 μm fluorogenic peptide substrate Z-Phe-Arg-7-amino-4-methylcoumarin (Z-FR-AMC; Enzo Life Sciences) in cathepsin assay buffer (100 mm sodium acetate, 120 mm NaCl, and 1 mm EDTA (pH 5.5)). Duplicate lysates were preincubated with 50 μm E64d, a membrane-permeable cysteine protease inhibitor, for 15 min at 37 °C to inhibit cathepsin enzyme activities. Fluorescence readings derived from Z-FR-AMC hydrolysis were measured with a Flexstation 3 (Molecular Device) for 60 min at 37 °C. Fluorescence measurements were collected with 380-nm excitation and 460-nm emission at 1-min intervals. The acid enzyme kinetics (*V*_max_) was calculated as changes in relative fluorescence unit/min.

##### Statistical Analysis

All data are expressed as means ± S.E. unless otherwise described. Statistical analyses were performed using Student's *t* test (unpaired and two-tailed). Analysis of variance was used for comparisons between more than two groups. A value of *p* < 0.05 was considered to be statistically significant.

## RESULTS

### 

#### 

##### Mice Lacking TPC2 Exhibit Muscle Atrophy but Not Muscular Dystrophy Phenotype

**FIGURE 1.**
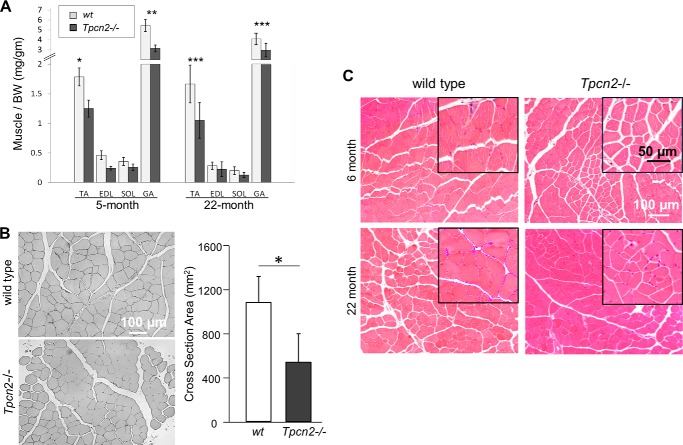
**Atrophic muscle phenotype of *Tpcn2*^−/−^ mice.** *A*, various skeletal muscle types were dissected from 5- and 22-month-old wild type and *Tpcn2*^−/−^ animals. The relative muscle weight to body weight (*BW*) was calculated. *Error bars* denote S.D. (*n* = 9 for 5-month-old mice; *n* = 6 for 22-month-old mice; *, *p* < 0.05; **, *p* < 0.01; ***, *p* < 0.001). *B*, representative cross-sections of the TA muscle from wild type and *Tpcn2*^−/−^ animals (5-months old) stained with anti-dystrophin to reveal myofiber boundaries. Quantification of the TA myofiber cross-sectional area is shown in the *bar chart*. Values are means ± S.E. (*n* = 4 mice for each group;*, *p* < 0.01). *C*, H&E staining of TA muscle from young (6-month-old) and aged (22-month-old) wild type (*left panels*) or *Tpcn2*^−/−^ (*right panels*) mice. No gross myopathy, such as central nuclei and fibrosis, was observed in *Tpcn2*^−/−^ muscles.

Although *Tpcn2*^−/−^ mice are viable, we found that they develop skeletal muscle atrophy. We compared the mass of four skeletal muscle types, including TA, extensor digitorum longus, soleus, and GA, from wild type and *Tpcn2*^−/−^ mice. Fig. 1*A* shows that the levels of a number of markers of muscle mass were significantly reduced in GA and TA at ages of 5 and 22 months. Based on anti-dystrophin immunostaining of the sarcolemmal membrane, the cross-sectional area of TA myofibers of 5-month-old *Tpcn2*^−/−^ mice was reduced by 46% when compared with wild type controls (Fig. 1*B*).

To explore further the role of TPC2 in muscle physiology, we examined, using H&E staining, TA muscle morphology from young (6-month-old) and aged (22-month-old) mice (Fig. 1*C*). Despite the fact that *Tpcn2*^−/−^ mice exhibited a general decrease in myofiber size, there was no evidence of gross myopathy, such as central nuclei typically found in dystrophic muscle. Thus, although the *Tpcn2*^−/−^ muscles show atrophy, they do not display any obvious muscular dystrophy.

##### Mice Lacking TPC2 Display Reduced Muscle Endurance, Altered Autophagy, and Lysosome Degradation

**FIGURE 2.**
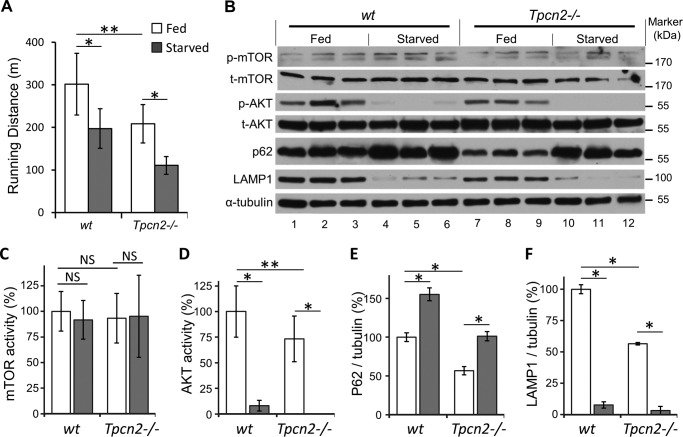
**Reduced endurance and altered autophagic signaling in skeletal muscle of *Tpcn2*^−/−^ mice.** *A*, mice (male; 4–5 months old) were tested on a treadmill under dietary conditions of regular chow or 3-day fasting. The distances traveled (in meters) until exhaustion were recorded and analyzed (*n* = 6 mice/group). Data are means ± S.E. (*, *p* < 0.05; **, *p* < 0.01). *B*, autophagy flux studies under regular diet or extended starvation. Mice (male; 4–5 months old) were fed with regular chow or fasted for 3 days. The TA muscles were then harvested and homogenized for immunoblotting with p-mTOR, total mTOR (*t-mTOR*), p-AKT, total AKT (*t-AKT*), p62, LAMP1, and α-tubulin. *C–F*, densitometry quantification of mTOR activity (p-mTOR/total mTOR; *C*), AKT activity (p-AKT/total AKT; *D*), normalized (over α-tubulin) p62 (*E*), and LAMP1 (*F*). *Error bars* represent S.E. (*n* = 6 mice/group; *, *p* < 0.05; **, *p* < 0.01; *NS*, not significant; analysis of variance).

Previously, *Tpnc1*/*Tpnc2* double knock-out mice were reported to perform poorly in the treadmill test during starvation (22). Because *Tpcn2* knock-out alone exhibited muscle atrophy, we considered the possibility that lack of TPC2 could be responsible for the reduced endurance. As in the previous study (22), we used a long fasting protocol; animals were subjected to either 3-day fasting or normal diet and then tested for treadmill endurance. As shown in Fig. 2*A*, even with a normal diet the treadmill test showed that *Tpcn2*^−/−^ mice ran shorter distances than wild type controls before exhaustion (302 ± 73 m for wild types, 209 ± 45 m for *Tpcn*2^−/−^, *n* = 6, *p* < 0.01). With starvation, both genotypes showed reduced endurance, but the endurance of *Tpcn2*^−/−^ mice was reduced by ∼50%, and that of wild type was reduced by only ∼30% (197 ± 46 m for wild types, 111 ± 21 m for *Tpcn*2^−/−^, *p* < 0.05 *versus* normal diet). Therefore, the TPC2 knock-out mice have decreased muscle endurance as compared with wild type no matter whether they are fed or starved.

**FIGURE 3.**
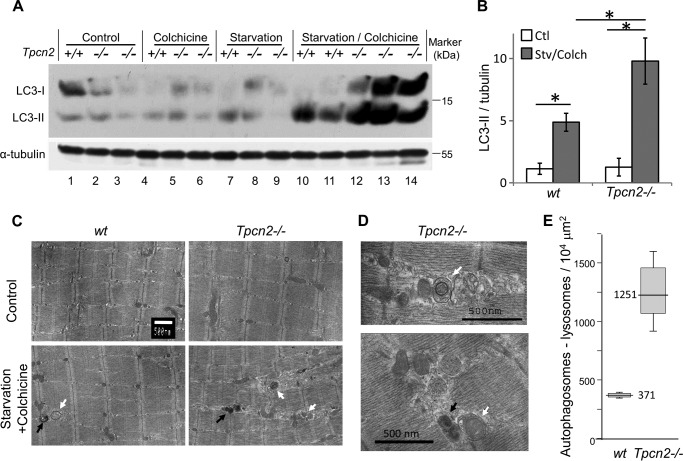
**Exacerbated autophagosome accumulation in *Tpcn2*^−/−^ skeletal muscle in response to microtubule inhibitor during starvation.** *A* and *B*, immunoblots of LC3 and α-tubulin in TA muscle homogenates (100 μg/lane) of 5-month-old wild type (+/+) and *Tpcn2*^−/−^ (−/−; as denoted on the *top* of the *lanes*) mice under regular fed condition (control; *lanes 1–3*), after a 2-day treatment with either colchicine alone (*lanes 4–6*) or starvation alone (*lanes 7–9*), or a combination of starvation plus colchicine (*lanes 10–14*). *B*, densitometry quantification of normalized LC3-II levels (over tubulin) under the control condition (*Ctl*) or after starvation/colchicine treatment (*Stv/Colch*). *Error bars* represent S.E. *C*, electron micrographs of TA muscles derived from wild type and *Tpcn2*^−/−^ mice. The *scale bar* represents 500 nm. An apparent increase in the number of autophagosomes (*white arrows*) and lysosomes (*black arrows*) was observed in the *Tpcn2*^−/−^ TA muscle after starvation/colchicine treatment. *D*, enlarged electron micrographs from autophagic *Tpcn2*^−/−^ TA muscle showing autophagosome (*top panel*, *white arrow*) and mitophagy (*lower panel*, *white arrow*). The lysosome is marked with a *black arrow. E*, the numbers of apparent autophagosomes plus lysosomes derived from 10–12 muscle cells/sample in wild type and *Tpcn2*^−/−^ TA following starvation/colchicine-induced autophagy. Data are expressed as numbers of autophagosomes plus lysosomes over the area of 10^4^ μm^2^. *Error bars* represent S.E. (*n* = 4 mice/condition).

To relate the endurance defect and muscle atrophy to autophagy, we investigated whether the critical energy mediators and autophagy-related signal molecules, such as mTOR, AKT, p62/SQSTM1, LAMP1, and LC3, were changed under both control and starvation stress conditions in TA muscles from wild type and *Tpcn2*^−/−^ animals (Figs. 2, *B–F*, and 3). Interestingly, the expression level of the mTOR core protein did not show a significant difference under either of these conditions (Fig. 2*C*). However, *Tpcn2*^−/−^ mice demonstrated significantly lower (−27%) AKT activity (p-AKT/total AKT; Fig. 2*D*) under a regular diet. Under fasting conditions, however, the p-AKT activity was dramatically decreased in both strains with wild type exhibiting only 8% of fed control and *Tpcn2*^−/−^ mice exhibiting almost complete loss of activity. This dramatic effect of starvation on AKT, but not mTOR, activity is consistent with previous findings that identify AKT kinase as a primary regulator of autophagy in skeletal muscle (11, 17).

p62/SQSTM1 is a selective autophagy substrate, and it acts as a ubiquitin-binding adaptor protein, which mediates recruitment of ubiquitinated protein cargos, via its interaction with LC3-II, into the inner membrane of autophagosome for selective autophagic degradation (35). As expected from the autophagic response to nutrient deprivation, the levels of p62/SQSTM1 in muscles from fasted wild type and *Tpcn2*^−/−^ mice were both elevated when compared with those fed with the regular diet (Fig. 2*E*). However, comparison of wild type and *Tpcn*2^−/−^ muscles identified a significant reduction (∼43%) of p62/SQSTM1 under fed conditions in mice lacking *Tpcn2*. Coincidentally, under the normal dietary conditions, the level of LAMP1, a predominantly lysosome-resident protein, was also reduced by ∼43% in *Tpcn*2^−/−^ muscles when compared with wild type (Fig. 2*F*), suggesting that basal autophagy flux in the *Tpcn*2^−/−^ muscles might be faster or more efficient than in wild type muscles. After starvation, LAMP1 expressions were reduced to comparable levels in both wild type (∼8%) and *Tpcn2*^−/−^ muscles (∼6%; Fig. 2*F*).

##### Skeletal Muscle of TPC2 Knock-out Mice Exhibit Increased Autophagy Flux

The term “autophagy flux” is used to represent the entire dynamic autophagic process, including autophagosome formation, maturation, autophagosome-lysosome fusion, macromolecule digestion, and lysosome recycling. During this process, LC3 proteins are specifically cleaved at the carboxyl terminus to form LC3-I, which is then conjugated with phosphatidylethanolamine (lipidated) to form LC3-II and becomes concentrated on autophagosome membranes. The LC3-II content therefore provides a good correlate of autophagic vesicle number (32, 36). However, changes in the LC3-II levels may result from alterations in the rate of either autophagosome synthesis or autophagosome turnover. We explored this process further and determined the impact of TPC2 deletion on outcomes. To this end, we exposed animals to either a normal diet, 2-day fasting, colchicine (0.4 mg/kg of body weight, intraperitoneal daily for 2 days) alone, or 2-day fasting plus colchicine following a previously published method (29). Colchicine was used to inhibit microtubule polarization and thereby to block the autophagosome-lysosome fusion and degradation of autophagosome content, including LC3-II. Although colchicine alone did not significantly change the LC3-II level in TA muscles from either wild type or *Tpcn*2^−/−^ mice, combined treatment with both 2-day starvation and colchicine resulted in marked accumulation of LC3-II in muscles from both strains with the increase in *Tpcn*2^−/−^ muscles being about twice that of wild types (Fig. 3, *A* and *B*). Noticeably, starvation plus colchicine treatment also increased the level of LC3-I in *Tpcn*2^−/−^ muscles but decreased it in wild type muscles (Fig. 3*A*). This would be consistent with an enhanced autophagosome synthesis, which caused generation of more LC3-I in the mutant muscles, although we cannot rule out a limit in the lipidation or an enhanced delipidation due to the overwhelming accumulation of LC3-II in TPC2-deficient cells. Also noticeable is that under our experimental conditions colchicine treatment alone did not significantly increase LC3-II levels in wild type muscles, which differs from the previous study (29). We suspect that dietary difference, environmental factors, and stress due to shipment prior to experimentation as in the case of the previous study may contribute to such a difference. However, because the colchicine treatment is intended to facilitate detection of LC3 proteins under starvation stress conditions (29), the lack of colchicine effect under fed conditions and its robust effect under starvation only suggest a well controlled experimental condition for assessing the starvation effect on autophagy flux in the current study. Because the increases in LC3-I and LC3-II levels were only detected under starvation when autolysosome formation was inhibited by colchicine, these data suggest that both autophagosome synthesis and turnover occur at faster rates or more frequently in *Tpcn*2^−/−^ than in wild type muscles, implicating a role for TPC2 in down-regulation of autophagy. This also supports the above notion that autophagy flux may be more efficient in *Tpcn2*^−/−^ cells based on reduced LAMP1 levels under fed conditions.

Examination of the ultrastructure of starved/colchicine treated TA skeletal muscle by EM revealed an increased number and abnormal distribution of autophagosomes/lysosomes. In this situation of enhanced autophagy, we also observed clear evidence of mitophagy as indicated by the presence of swollen and damaged mitochondria wrapped by autophagosomes in the TA muscle of the *Tpcn2*^−/−^ mice (Fig. 3, *C* and *D*). Quantitative analysis indicated a significant increase in the density of autophagosomes/lysosomes in *Tpcn2*^−/−^ muscle (1251 ± 351/10^4^ μm^2^) as compared with the wild type muscle (371 ± 19/10^4^ μm^2^; Fig. 3*E* and Table 1). Thus, the results from EM analysis support the conclusion drawn from immunoblotting LC3 proteins, that autophagosome formation was enhanced in *Tpcn*2^−/−^ muscles under combined starvation and colchicine treatment. Together, our results demonstrate that, in the presence of a microtubule inhibitor to block vesicle trafficking during autophagy induction, there was enhanced accumulation of lipidated LC3 and its intermediate form LC3-I and increased density of autophagosomes and lysosomes in skeletal muscle of *Tpcn2*^−/−^ mice indicative of an inhibitory role of TPC2 in autophagy.

**TABLE 1:** **Appearance of autophagosomes and lysosomes from muscle autophagy flux studies: rough comparison of electron micrographs** The appearance of autophagosomes and lysosomes from each muscle specimen was blindly examined and roughly compared (− or +/−, no induction; +, slight induction; ++ or ++++, induction). The numbers of autophagosomes plus lysosomes for starved plus colchicine treatment were further investigated and are summarized in Fig. 3*E*.

	Control	Starved	Colchicine	Starved + colchicine
WT	− or +/−	+/−	− or +/−	++
*Tpcn2*^−/−^	+/−	+	+	++++

##### Increased Autophagy Flux in Cultured Myotubes from Tpcn2^−/−^ Mice

Using a similar approach, we assayed autophagy flux in cultured myotubes. Myoblasts were isolated from wild type and *Tpcn2*^−/−^ neonates, differentiated into myotubes, and then subjected to autophagy flux analysis. We applied Baf A1, a potent and specific vacuolar H^+^-ATPase proton pump inhibitor, which prevents autophagosome maturation into autolysosomes by inhibiting lysosomal acidification and in turn blocking the fusion of autophagosomes and lysosomes (37). Under these conditions, the accumulation of LC3-II reflects autophagosome synthesis in the absence of turnover (degradation).

**FIGURE 4.**
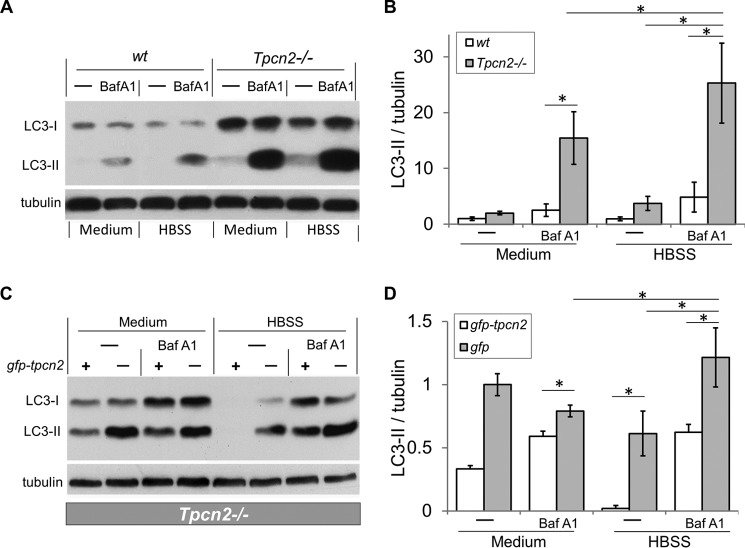
**Exacerbated autophagosome accumulation in cultured myotubes from *Tpcn2*^−/−^ mice under nutrient deprivation and inhibition of vacuolar H^+^-ATPase.** *A*, differentiated myotubes derived from the wild type or *Tpcn2*^−/−^ neonates were subjected to autophagy flux measurement (see “Experimental Procedures”). Baf A1 at concentration of 200 nm was used to inhibit the vacuolar H^+^-ATPase in the lysosome. HBSS was used to induce starvation of the cells. Cell lysates (40 μg of proteins/lane) were used for immunoblotting with anti-LC3 or anti-α-tubulin. *B*, relative levels of LC3-II/tubulin. Data are expressed as -fold induction relative to that of the wild type at basal condition (medium) (*n* = 4 experiments). Data are means ± S.E. (*, *p* < 0.01). *C*, *Tpcn2*^−/−^ myoblasts were transfected with either *gfp-tpcn2* plasmid (*odd-numbered lanes*) or pcms-eGFP plasmid (*even-numbered lanes*) for 48 h and then subjected to autophagy flux analysis. Cell lysates (30 μg of proteins/lane) were collected for immunoblots with anti-LC3 or anti-α-tubulin. *D*, relative levels of LC3-II/tubulin. Data (means ± S.E.) are expressed as LC3-II/tubulin normalized to that obtained from pcms-eGFP-transfected *Tpcn2*^−/−^ cells cultured under basal conditions (medium; *lane 2*) (*n* = 3 experiments). *Error bars* represent S.E.

As shown in Fig. 4*A*, LC3-I levels were markedly higher in *Tpcn2*^−/−^ than in wild type myotubes regardless of whether the cells were medium-fed or starved in HBSS. This occurred even without inhibiting autolysosome formation using Baf A1, indicating that most likely the cultured *Tpcn2*^−/−^ muscles also exhibited enhanced autophagosome synthesis just like the starved muscles *in vivo*. In nutrient-deprived cultures (grown in HBSS), the LC3-II level was significantly higher in *Tpcn2*^−/−^ than in wild type myotubes, and Baf A1 treatment increased LC3-II levels in both wild type and *Tpcn2*^−/−^ myotubes whether medium-fed or nutrient-deprived. However, the LC3-II levels were significantly greater in *Tpcn2*^−/−^ than in wild type myotubes under all conditions (Fig. 4, *A* and *B*). These results support the finding from intact muscles *in vivo* that autophagosome synthesis is increased in the absence of TPC2. The finding that LC3-II levels remained largely unaltered in the absence of inhibited autophagosome turnover in both *in vivo* and *in vitro* models suggests that increased synthesis was matched by an equivalent rate of autolysosome formation in TPC2-null cells. Therefore, the *Tpcn2* deletion resulted in enhanced autophagy flux in mouse skeletal muscle. Notably, the increased LC3 proteins found in *Tpcn2*^−/−^ muscles could not be simply accounted for by a change in its transcription as the analysis of LC3 mRNA level by quantitative real time PCR did not reveal a significant difference between muscles from wild type and *Tpcn2*^−/−^ mice (data not shown).

To confirm that the lack of TPC2 rather than other compensatory effects that potentially occurred in the *Tpcn2*^−/−^ muscles was the cause for the enhanced autophagy flux, we expressed either GFP (as control) or GFP-TPC2 fusion protein in *Tpcn2*^−/−^ myoblasts and then performed the autophagy flux assay. As shown in Fig. 4, *C* and *D*, complementing *Tpcn2* deletion with GFP-TPC2 reduced LC3-II formation in medium-fed and nutrient-starved conditions with or without Baf A1 treatment, indicating that TPC2 plays a critical role in reducing autophagy flux in myoblasts. Note, in Fig. 4*D*, the LC3-II/tubulin values were normalized to that of control (GFP-transfected) *Tpcn2*^−/−^ myocytes. The reversal by GFP-TPC2 expression was partial because the transfection efficiency was about 30–40%.

##### Loss of TPC2 Leads to Abnormal Lysosomal pH and Impaired Acid Enzymatic Activity in Skeletal Muscle

**FIGURE 5.**
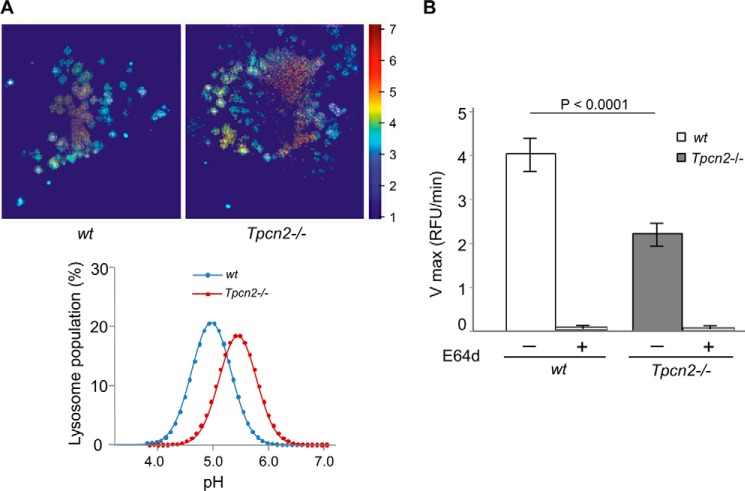
**TPC2-null skeletal muscle exhibits altered intralysosomal pH and impaired lysosomal acid enzyme activity.** *A*, lysosomal pH in wild type and *Tpcn2* knock-out myoblasts. Wild type and *Tpcn2*^−/−^ myoblasts were isolated, and the lysosomal pH was measured by pH-sensitive ratiometric imaging of Oregon Green 514-labeled dextran taken up by myoblasts under regular medium conditions. Representative Oregon Green 514-dextran labeled lysosomes from wild type (*top left*) and *Tpcn2*^−/−^ (*top right*) myoblasts are shown. The histograms of the lysosomal pH distribution values (fitted to a Maxwell-Boltzmann curve) are shown (*bottom*). The means ± S.E. from three experiments are 4.97 ± 0.32 for wild type and 5.40 ± 0.23 for *Tpcn2*^−/−^. *B*, acid protein extracts derived from GA muscles of wild type (*wt*) or *Tpcn2* ^−/−^ mice (6-months old) were used to measure the cathepsin protease kinetics with 20 μm Z-FR-AMC fluorogenic peptide as the substrate. Specific cathepsin activity was assessed using the cysteine protease inhibitor E64d. The *V*_max_ for wild type muscle is 4.03 ± 0.38, whereas for *Tpcn2*^−/−^ muscle, *V*_max_ is 2.20 ± 0.26. Values are means ± S.E. (*n* = 6 mice). *Error bars* represent S.E. *RFU*, relative fluorescence units.

To determine whether TPC2 regulates lysosomal pH homeostasis and enzymatic activity in skeletal muscle, the *Tpcn2*^−/−^ myoblasts were used to assay for evidence of homeostatic changes in lysosome function relative to wild type. First, we examined luminal pH with a ratiometric analysis of lysosomes loaded with dextran-conjugated Oregon Green 514 (33) in myoblasts. As shown in Fig. 5*A*, wild type lysosomes maintained a mean pH of 4.97 ± 0.32, whereas *Tpcn2*^−/−^ myoblasts exhibited a more alkaline lysosome pH of 5.40 ± 0.21 under basal conditions. Thus, in skeletal muscle lysosomes, TPC2 appears to contribute to luminal pH homeostasis and possibly the capacity of lysosomes to resist evoked changes in luminal pH.

Second, dysfunction of pH regulation would likely impair the activity of lysosome-resident enzymes. Among them, the cathepsin family proteases are expressed and activated in the acidic compartment of lysosomes (38). We therefore assessed the kinetics of lysosomal protease isolated from acidic extracts of GA muscle to hydrolyze an OmniCathepsin fluorogenic peptide substrate, Z-FR-AMC (Fig. 5*B*). The *V*_max_ of lysosomal protease from skeletal muscle of *Tpcn2*^−/−^ mice was half that of lysosomal protease isolated from wild type skeletal muscle, suggesting impaired lysosomal protease activities in skeletal muscles that lack TPC2.

##### The Lack of TPC2 in Skeletal Muscle Impairs Autophagy Termination

**FIGURE 6.**
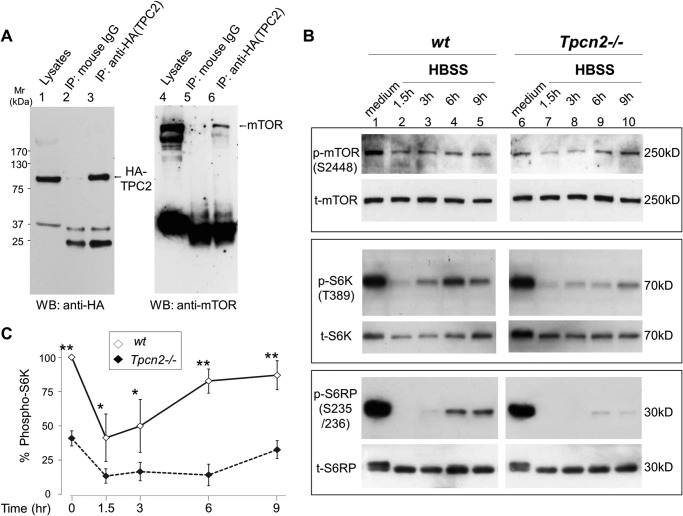
**Reduced mTOR activity and delayed mTOR reactivation during starvation in cultured myotubes from *Tpcn2*^−/−^ mice.** *A*, physical association between TPC2 and mTOR in skeletal muscle. TA muscle transfected with HA-TPC2 was used for a co-immunoprecipitation (*IP*) assay to test the association between HA-TPC2 and mTOR. Immunoprecipitation was performed with anti-HA (12CA5) antibody or with mouse IgG as a control. The *left panel* shows an immunoblot with anti-HA, and the *right panel* shows an immunoblot with anti-mTOR. *B*, myotubes derived from wild type or *Tpcn2*^−/−^ were subjected to HBSS starvation treatment for different time periods as denoted. Cell lysates (40 μg/lane; *lanes 1–5*, wild type; *lanes 6–10*, *Tpcn2*^−/−^) were analyzed by immunoblotting of p-mTOR(Ser-2448) and total mTOR (*t-mTOR*) (*top panels*); p-S6K(Thr-389) and total S6K (*t-S6K*) (*middle panels*), and p-S6RP(Ser-235/236) and total S6RP (*t-S6RP*) (*bottom panels*). *C*, mTOR activities were compared based on the relative p-S6K (p-S6K/total S6K) levels. Data are expressed as percentage of p-S6K (p-S6K/total S6K) in relation to the value from wild type medium control. Values are means ± S.E. (*n* = 4 experiments; *, *p* < 0.05; **, *p* < 0.01). *Error bars* represent S.E. *WB*, Western blot.

Previously, mTOR was shown to physically associate with both TPC1 and TPC2 and inhibit their functions in HEK293T cells. We have confirmed the physical interaction between mTOR and TPC2 in TA skeletal muscle after electroporation-mediated delivery of HA-tagged TPC2 plasmid into the muscle tissue of viable wild type mice following our published procedures (30). Two weeks postelectroporation, abundant expression of HA-TPC2 was detected in the TA muscle, and co-immunoprecipitation analysis demonstrated that endogenous mTOR formed complexes with HA-TPC2 (Fig. 6*A*).

The localization of mTOR to lysosomes contributes to nutrient sensing and thus to the regulation of autophagy (21). The previous study has placed TPCs downstream from mTOR signaling based on the findings that nutrient-driven translocation of mTOR to lysosomes and phosphorylation of p70S6 kinase-1 (S6K), a key downstream effector of mTOR in protein synthesis, were intact in cells from *Tpcn1*/*Tpcn2* double knock-out mice (22). However, upon examining the levels of phospho-mTOR (p-mTOR), phospho-S6K (p-S6K), and phospho-S6 ribosomal protein (p-S6RP) in cultured myotubes prepared from wild type and *Tpcn2*^−/−^ mice under fed and nutrient-deprived conditions (Fig. 6*B*), we found that the *Tpcn2*^−/−^ myotubes exhibited a markedly reduced (∼45% relative to wild type) mTOR kinase activity (based on the analysis of p-mTOR(Ser-2448), p-S6K, and p-S6RP activities) under basal conditions (Fig. 6, *B* and *C*). Furthermore, in wild type myotubes, mTOR kinase activity rebounded 6 h post-nutrient deprivation, indicating competent autophagic lysosomal nutrient recycling. By contrast, mTOR reactivation in *Tpcn2*^−/−^ myotubes was delayed until 9 h post-nutrient deprivation, and mTOR activity remained low relative to wild type at all time points analyzed (Fig. 6, *B* and *C*). These results therefore indicate that TPC2 deficiency is linked to reduced mTOR activity in myotubes, which may result from impairment in mTOR reactivation for autophagy termination in the continued absence of nutrients. This is entirely consistent with the generally accepted view that autophagy terminates during prolonged starvation in response to the nutrients generated from degradation of autolysosomal products and that mTOR is involved in this process (9).

##### Lack of TPC2 Expression Is Associated with Histopathological Phenotypes in Multiple Tissues during Aging

**FIGURE 7.**
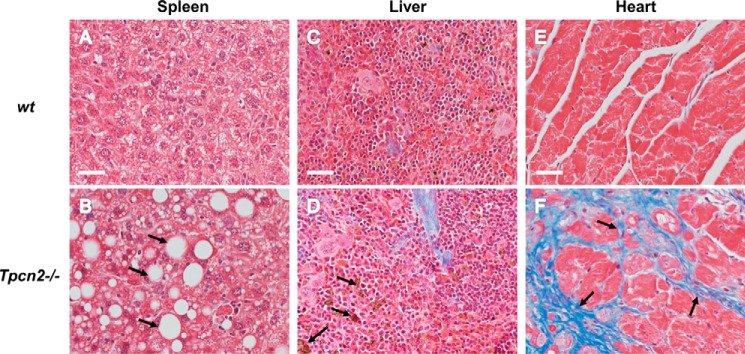
**Histopathological micrographs of aged tissues.** *A–F*, representative trichrome staining of spleen (*A* and *B*), liver (*C* and *D*), and heart (*E* and *F*) derived from 22-month-old wild type (*A*, *C*, and *E*) or *Tpcn2*^−/−^ (*B*, *D*, and *F*) mice. Abundant empty vacuoles (*B*, *arrows*) from spleen, lipofuscin deposits (*D*, *arrows*) from liver, and fibrosis (*F*, *arrows*) from heart were found in aged tissues from *Tpcn2*^−/−^ mice. *Scale bars*, 50 μm.

There is a close interplay between autophagy and aging (39). The age-related defects in protein turnover and accumulation of dysfunctional cellular components result in loss of cellular homeostasis. We examined aged tissues with high autophagy flux to verify whether TPC2 regulation on autophagy had any role in other tissues (Fig. 7). We found that spleens from aged *Tpcn2*^−/−^ mice were full of large vacuoles; their livers contained high levels of lipofuscin, an insoluble aggregate of oxidized proteins and lipids; and their cardiomyocytes demonstrated enhanced fibrosis. In summary, these suggest multiple defects in cellular homeostasis in aged *Tpcn2*^−/−^ tissues.

## DISCUSSION

In this study, we report a skeletal muscle atrophy phenotype in mice lacking TPC2 expression, which differs from the previous report showing no obvious defect in either muscle or autophagy regulation in the TPC1/TPC2 double KO mice (22). This discrepancy could result from the different strategies used in generating the TPC2 knock-out mice. In the report by Cang *et al.* (22), the *Tpcn2* gene was disrupted by deleting the coding sequence for the first 49 amino acids of the TPC2 protein, which could lead to a truncated product with partial function. By contrast, the *Tpcn2*^−/−^ mice used in the current study were generated by a gene trap technique with the deletion of exons 4–9 in the middle of the transcript. In addition, variations in the background stains, diets, and animal husbandry might also contribute to the differences between the two studies.

We detected increases in autophagic flux under starvation in both cultured myotubes and intact muscle tissues derived from the *Tpcn2*^−/−^ mice as shown by the dramatic increase in LC3-II levels when autolysosome formation was blocked by treatment with either Baf A1 to disrupt the lysosome pH gradient in the cultured cells or colchicine to impair lysosome trafficking in the skeletal muscle of live animals. The increase in the LC3-II levels was reverted by reintroducing human TPC2 into the *Tpcn2*^−/−^ muscle. Importantly, with the loss of TPC2, mTOR kinase activity in cultured myotubes was significantly decreased, and reactivation of mTOR during prolonged starvation was severely delayed, implicating a new role for TPC2 in mTOR reactivation and autophagy termination.

A previous study demonstrated physical interaction and functional coupling between mTOR and TPCs (22). However, whether and how TPCs affect mTOR function and in turn regulate autophagy remained unclear. Of the two TPCs, TPC2 is more preferentially localized on lysosomal membranes than TPC1 (23). Despite the significant atrophy, the *Tpcn2*^−/−^ skeletal muscle did not show obvious accumulation of the autophagy markers p62/SQSTM1 and LC3 under normal conditions. Rather the level of p62/SQSTM1 was reduced in *Tpcn2*^−/−^ muscle as compared with the wild type tissue. This reduction could reflect a more efficient autophagy flux that degrades p62 (40) in *Tpcn2*^−/−^ muscle (Fig. 2*B*). Interestingly, the loss of TPC2 also appears to lead to a homeostatic change that affects the overall content and activity of lysosomes in *Tpcn2*^−/−^ skeletal muscle under basal conditions as shown by reduction of the LAMP1 level (Fig. 2, *B* and *F*) and decreased cathepsin activity (Fig. 5*B*). At present, the mechanism that led to the reduced enzymatic activity for cathepsin in the *Tpcn2*^−/−^ muscle remains unclear. It is possible that the altered homeostatic capacity for pH (Fig. 5*A*) in the *Tpcn2*^−/−^ lysosome may contribute to the defective folding and/or processing of the cathepsin enzymes, which together may underlie the defective lysosome function associated with autophagy signaling. The dramatic loss of LAMP1 in response to starvation in both wild type and *Tpcn2*^−/−^ muscles indeed suggests utilization of the lysosome reserve for autolysosome formation and degradation during autophagy in skeletal muscle. Therefore, a decrease in the lysosome reserve may be anticipated in the *Tpcn2*^−/−^ muscle if basal autophagic flux is augmented.

Conversely, a change in LC3-II level was not obvious in the *Tpcn2*^−/−^ muscle obtained from fed mice, and starvation alone did not result in a dramatic change in LC3-II level unless lysosome trafficking was inhibited by colchicine. Thus, autophagosome fusion with lysosomes must be intact and efficient enough to match the enhanced autophagosome synthesis in *Tpcn2*^−/−^ even under starved conditions. The question arises as to why colchicine treatment alone did not cause LC3-II accumulation in muscles from fed *Tpcn2*^−/−^ mice if the basal autophagy flux was enhanced. It is possible that the amount of colchicine (0.4 mg/kg) administered might be insufficient to abolish autolysosome formation and that the residual functional lysosomes have the necessary capacity to fuse with autophagosomes and degrade their contents under the basal conditions. However, we were unable to test a higher colchicine dose (*e.g.* 0.6 mg/kg) because it became lethal to the mice. Nevertheless, Baf A1 treatment in the normal medium did strongly enhance LC3-II accumulation in cultured myotubes, demonstrating enhanced autophagy flux in *Tpcn2*^−/−^ muscle under basal conditions. Again, without disrupting lysosome function with Baf A1, the levels of LC3-II were low in both wild type and *Tpcn2*^−/−^ mice irrespective of nutrient availability, indicating efficient autophagosome turnover in both genotypes.

How does TPC2 deficiency result in enhanced autophagy flux? One reason could be an impaired pH regulation in the lysosome lumen as shown for macrophages from *Tpcn1*/*Tpcn2* double knock-out mice (22). Our examination of luminal pH using ratiometric analysis of lysosomes loaded with dextran-conjugated Oregon Green 514 (33) in myoblasts indicated that wild type lysosomes maintained a mean pH of 4.97 ± 0.32, whereas *Tpcn2*^−/−^ myoblasts exhibited a more alkaline lysosome pH of 5.40 ± 0.21 under basal conditions (Fig. 5*A*). However, changes in luminal pH regulation do not necessarily lead to enhanced autophagy flux. With overexpression of TPC2 in HeLa cells, the lysosomal pH regulation was similarly altered, but the observed effects were associated with impaired autophagosome-lysosome fusion and LC3-II degradation (27). A second reason might be that the loss of TPC2 could alter mTOR function and in turn impair the autophagy cycle. Although we did not find a significant change in total mTOR, mTOR kinase activity was decreased in *Tpcn2*^−/−^ myotubes, which could be responsible for enhanced autophagy. This change is consistent with the detected decrease of phosphorylated AKT, which positively regulates mTOR, in intact muscle from the mutant *Tpcn2*^−/−^ mice. More importantly, mTOR (3) reactivation during prolonged starvation was delayed in the absence of TPC2, implicating a defect in autophagy termination. A third reason, as mentioned above, could be that autophagy terminates as a result of the recovery of key nutrients, including the major end products, amino acids, from the degradation of the autophagosome cargo in autolysosomes (9). Amino acid deficiency is the major cause of autophagy. Although amino acid sensing of mTOR complex 1 has been suggested to occur at a lysosomal luminal site (41), the availability of newly generated amino acids in the cytosol is still essential for protein synthesis. For this reason, the suggested function of TPCs in facilitating amino acid release from lysosomes (22) is particularly intriguing as it implies impairment of substrate supply via autophagy for a new round of protein synthesis. This may likely be a major cause of delayed autophagy termination in *Tpcn2*^−/−^ muscle.

The apparent increase in autophagy flux in *Tpcn2*^−/−^ myoblasts and muscle tissues likely reflects a deficiency in autophagy termination because of the compromised amino acid export from lysosomes. This may occur at the expense of continued degradation of cellular content without serving the need of facilitating survival under stress, the cytoprotective role of autophagy (3, 4). Therefore, mice lacking TPC2 exhibit an atrophy phenotype in the skeletal muscle and poor endurance during excise. The fact that these changes occurred in well fed mice is consistent with the notion that skeletal muscles undergo basal autophagy to maintain proper mass and function (4). In other tissues, the exacerbated autophagy flux in *Tpcn2*^−/−^ mice may also result in abnormalities in protein quality control and morphological changes, especially with aging (39). The age-related defects in protein turnover and accumulation of dysfunctional cellular components result in loss of cellular homeostasis. Indeed, in aged tissues with high autophagy flux, we found histological abnormalities in *Tpcn2*^−/−^ mice, including large empty vacuoles in the spleen, high levels of lipofuscin in the liver, and enhanced fibrosis in cardiomyocyte (Fig. 7). Therefore, the enhanced autophagy flux in the absence of TPC2 is detrimental to cell survival.

Recent research highlights the likelihood that metabolic modulation of skeletal muscle mass involves the complex interplay between both the ubiquitin-proteasome system and the autophagy-lysosome system (2, 11, 15, 16). Therefore it is interesting that *Tpcn2*^−/−^ mice develop muscle atrophy without muscular dystrophy as the *Tpcn2*^−/−^ muscle showed no obvious phenotype of fibrosis and accumulation of central nuclei. This suggests that the primary muscle defect likely affects myofibril protein turnover and not the regenerative capacity of the muscle. Our data are therefore indicative of defective protein quality control and/or recycling function in the absence of TPC2 and of this being one contributing factor to the observed defects in metabolic function in skeletal muscle.

In light of recent reports showing that TPC2 overexpression caused LC3-II accumulation in astrocytes (26) and HeLa cells (27), our data support the role of TPC2 in autophagy regulation but suggest a different underlying mechanism. It was proposed that the lysosomal pH increase associated with TPC2 overexpression was responsible for accumulation of autophagosomes. However, similar pH increases were observed in cells from *Tpcn2*^−/−^ and *Tpcn1*/*Tpcn2* double knock-out mice, but these did not lead to significant increases in LC3-II levels under either fed or starved conditions. Therefore, the moderate neutralization of lysosomal luminal pH does not explain the failure of autophagosome degradation in TPC2-overexpressing cells. Deregulation of Ca^2+^ homeostasis due to enhanced NAADP/TPC signaling has also been suggested (26, 27), but exactly how this affects autophagosome and lysosome fusion remains to be elucidated. Given that our previous studies have shown that TPC2 supports NAADP-dependent Ca^2+^ release across the lysosomal membrane (23, 42), alterations in TPC2 expression and function will impact the movement of Ca^2+^ across the lysosome membrane and could thus affect fission and fusion events of lysosomes and autophagosomes and the execution of autophagy. A greater understanding of the role of lysosome Ca^2+^ movement and its modulation by TPC2 during autophagy will therefore provide important insights into the mechanisms of autophagy regulation.

TPC1 and TPC2 have also been proposed to act as ATP-sensitive Na^+^ channels that are modulated by mTOR in response to changes in metabolic state (22, 25). Consequently, as cellular ATP levels fall, mTOR disassociates from TPCs, leading to channel opening and the release of Na^+^ and other cations from the lysosome lumen (22). Moreover, several signaling pathways, including Mg^2+^, Na^+^, NAADP, phosphatidylinositol 3,5-bisphosphate, and multiple kinases, have been demonstrated to convergently regulate TPC2-mediated Ca^2+^ signaling (43). All these likely contribute to the importance of local phosphatidylinositol 3,5-bisphosphate synthesis and Ca^2+^ signaling to the regulation of endolysosomal trafficking and fusion (44, 45), and our findings provide further support for the idea that direct association between mTOR and TPC2 on the lysosome participates in nutrient-dependent control of autophagy flux in skeletal muscle. The disruption of this association in the absence of TPC2 could result in impaired nutrient sensitivity and decreased mTOR activity. Although we did not find a significant change in the total mTOR level, we showed a reduced AKT activity in *Tpnc2*^−/−^ muscles isolated from animals and decreased p-mTOR, p-S6K, and p-S6RP levels in cultured myotubes prepared from the mutant mice. Our study therefore suggests at least a partial TPC2- dependence of the PI3K/AKT/mTOR pathway and thus a role in the regulation of autophagy in skeletal muscle. This agrees with the notion that AKT plays a major role in modulating skeletal muscle autophagy, possibly through its transcription-dependent regulation of FoxO3 (11, 17). We propose that TPCs are involved in the response to metabolic status and subsequent adaptive changes to the endolysosome system, which is especially important in skeletal muscle because of the plasticity of its energy status.

Autophagy has not only been indicated in the control of muscle mass (12, 46) but also in the aging process (39). In this respect, our studies on aged animals also support the idea that TPC2 affects homeostasis across a wide variety of tissues during aging (Fig. 7). That this is the case is perhaps not surprising given that defective autophagy has been shown to be involved in several lysosomal myopathies, such as lysosomal glycogen storage disease type II (47), Danon myopathy (48), X-linked myotubular myopathy (49), valosin-containing protein myopathy (50), and Jumpy mutation-central nucleus myopathy (51). Reactivation of autophagy has therefore emerged as a therapeutic strategy and has proved beneficial to some myopathological diseases, for example Ulrich myopathy (52) and Duchenne muscular dystrophy (53). Therefore, the mechanisms delineated in this study not only make a significant contribution to our developing understanding of autophagy regulation but also identify new therapeutic targets. However, further research is still needed to fully unravel the complex interplay between autophagic pathways in health and disease.

## Footnotes

3 The abbreviations used are:

AKT: protein kinase B
Baf A1: bafilomycin A1
GA: gastrocnemius
TA: tibialis anterior
LAMP: lysosome-associated membrane protein
LC3: microtubule-associated protein 1A/1B light chain 3A
mTOR: mammalian target of rapamycin
NAADP: nicotinic acid adenine dinucleotide phosphate
S6K: ribosomal S6 kinase
S6RP: S6 ribosomal protein
TPC: two-pore channel
eGFP: enhanced GFP
HBSS: Hanks' balanced saline solution
AMC: amino-4-methylcoumarin
Z: benzyloxycarbonyl
SQSTM1: sequestosome-1
p-AKT: phosphorylated AKT.
